# UK Biobank: An Open Access Resource for Identifying the Causes of a Wide Range of Complex Diseases of Middle and Old Age

**DOI:** 10.1371/journal.pmed.1001779

**Published:** 2015-03-31

**Authors:** Cathie Sudlow, John Gallacher, Naomi Allen, Valerie Beral, Paul Burton, John Danesh, Paul Downey, Paul Elliott, Jane Green, Martin Landray, Bette Liu, Paul Matthews, Giok Ong, Jill Pell, Alan Silman, Alan Young, Tim Sprosen, Tim Peakman, Rory Collins

**Affiliations:** 1 University of Edinburgh, Edinburgh, United Kingdom; 2 UK Biobank, Stockport, United Kingdom; 3 University of Cardiff, Cardiff, United Kingdom; 4 University of Oxford, Oxford, United Kingdom; 5 University of Bristol, Bristol, United Kingdom; 6 University of Cambridge, Cambridge, United Kingdom; 7 Imperial College, London, United Kingdom; 8 University of New South Wales, Sydney, Australia; 9 University of Warwick, Warwick, United Kingdom; 10 University of Glasgow, Glasgow, United Kingdom; 11 University of Manchester, Manchester, United Kingdom

## Abstract

Cathie Sudlow and colleagues describe the UK Biobank, a large population-based prospective study, established to allow investigation of the genetic and non-genetic determinants of the diseases of middle and old age.

**Summary Points**

- UK Biobank is a very large and detailed prospective study with over 500,000 participants aged 40–69 years when recruited in 2006–2010.
- The study has collected and continues to collect extensive phenotypic and genotypic detail about its participants, including data from questionnaires, physical measures, sample assays, accelerometry, multimodal imaging, genome-wide genotyping and longitudinal follow-up for a wide range of health-related outcomes.
- Wide consultation; input from scientific, management, legal, and ethical partners; and industrial-scale, centralised processes have been essential to the development of this resource.
- UK Biobank is available for open access, without the need for collaboration, to any bona fide researcher who wishes to use it to conduct health-related research for the benefit of the public.

The challenge of understanding the determinants of common life-threatening and disabling conditions is substantial. These conditions are typically caused by a combination of lifestyle, environmental, and genomic factors, with individually modest effects and complex interactions, the detection and quantification of which require studies with large numbers of disease cases. While retrospective case-control studies of particular diseases [1] or existing prospective studies of particular risk factors can help to address this challenge [2,3], a complementary approach is to establish large prospective cohorts designed to study a much wider range of known and novel risk factors for a wide range of diseases [4]. Prospective studies can assess exposures before the onset and treatment of disease, diseases that are not readily investigated by retrospective studies, and both the adverse and beneficial effects of a specific exposure on the lifetime risks of different diseases.

UK (United Kingdom) Biobank is a very large, population-based prospective study, established to allow detailed investigations of the genetic and nongenetic determinants of the diseases of middle and old age [5,6]. It aims to combine extensive and precise assessment of exposures with comprehensive follow-up and characterisation of many different health-related outcomes, as well as to promote innovative science by maximising access to the resource. Recruitment of 500,000 participants and the collection of an unprecedented wealth of baseline data and samples were completed in 2010. Activity is now focused on further phenotyping of participants and their health outcomes and on providing access to researchers from around the world.

## Cohort Size

The large size of the cohort was based on statistical power calculations for nested case-control studies [7], showing that 5,000–10,000 cases of any particular condition would be required for the reliable detection of odds ratios (ORs) for the main effects of different exposures of 1.3–1.5 (the upper end of the range reported from genome-wide association studies of various conditions [8]), and around 20,000 cases for detection of interactions with ORs of at least 2.0. To observe such large numbers of cases of particular diseases within a reasonable follow-up period, prospective cohorts need very large numbers of participants. Projected numbers of cases of a range of common conditions expected to occur among 500,000 UK Biobank participants during 20 years of follow-up (Table 1) suggest that reliable assessment of the main determinants of most of these conditions (and others that are similarly common) should be possible during the current decade [6,9]. The age range for inclusion of 40–69 years represented a pragmatic compromise between participants being old enough for there to be sufficient incident health outcomes during the early years of follow-up and young enough for the initial assessment to occur before incipient disease had a material impact on exposures.

**Table 1:** Approximate numbers of incident cases of some exemplar conditions expected to accrue during the first 20 years of follow-up in UK Biobank.

Condition	2012	2017	2022	2027
Diabetes mellitus	10,000	25,000	40,000	68,000
MI and coronary death	7,000	17,000	28,000	47,000
Stroke	2,000	5,000	9,000	20,000
COPD	3,000	8,000	14,000	25,000
Breast cancer (female)	2,500	6,000	10,000	16,000
Colorectal cancer	1,500	3,500	7,000	14,000
Prostate cancer	1,500	3,500	7,000	14,000
Lung cancer	800	2,000	4,000	8,000
Hip fracture	800	2,500	6,000	17,000
Rheumatoid arthritis	800	2,000	3,000	5,000
Alzheimer’s disease	800	3,000	9,000	30,000
Parkinson’s disease	1000	3,000	6,000	14,000

Based on UK age- and sex-specific rates with adjustment for healthy cohort effects and losses to follow-up [6].

MI: myocardial infarction; COPD: chronic obstructive pulmonary disease or chronic bronchitis/emphysema

## Data Availability

### Data from the Baseline Assessment

The 500,000 participants were assessed between 2006 and 2010 in 22 assessment centres throughout the UK, covering a variety of different settings to provide socioeconomic and ethnic heterogeneity and urban–rural mix. This ensured a broad distribution across all exposures to allow the reliable detection of generalisable associations between baseline characteristics and health outcomes. The assessment visit comprised electronic signed consent; a self-completed touch-screen questionnaire; brief computer-assisted interview; physical and functional measures; and collection of blood, urine, and saliva (Table 2). Multiple aliquots of different sample fractions are stored in UK Biobank’s automated laboratory, allowing for a wide range of future assays [10].

**Table 2:** Data collected at the baseline assessment.

Questionnaire and interview	
Sociodemographic	Social class; ethnicity; employment status; marital status; education; income; car ownership
Family history and early life exposures	Family history of major diseases; birth weight; breast feeding; maternal smoking; childhood body size; residence at birth
Psychosocial factors	Neurosis; depression (including bi-polar spectrum disorder); social support
Environmental factors	Current address; current (or last) occupation; domestic heating and cooking fuel; housing; means of travel; shift work; mobile phone use; sun exposure
Lifestyle	Smoking; alcohol consumption; physical activity; diet; sleep
Health status	Medical history; medications; disability; hearing; sight; sexual and reproductive history
Hearing threshold	Speech reception threshold*
Cognitive function	Pairs matching; reaction time; prospective memory*; fluid intelligence*; numeric memory^†^

Physical measures	
Blood pressure and heart rate	two automated measures, one minute apart
Grip strength	Left- and right-hand grip strength
Anthropometrics	Standing and sitting height; weight and bio-impedance; hip and waist circumference
Spirometry	Up to three measures
Bone density^‡^	Calcaneal ultrasound
Arterial stiffness^¶^	Pulse wave velocity
Eye examination^§^	Refractive index, intraocular pressure; acuity; retinal photograph; optical coherence tomography
Fitness test^§^	Cycle ergometry with electrocardiogram (ECG) heart rate monitoring

* assessed in 170,000 participants;

^†^ assessed in 50,000 participants;

^‡^measured in one heel for 170,000 participants and in both heels for 320,000 participants;

^¶^ measured in 170,000 participants;

^§^ measured in 100,000 participants

### Data from Additional Assessments to Enhance Phenotyping

UK Biobank is conducting a range of additional phenotyping assessments in all (or large subsets) of the participants. Data are already available both from a detailed dietary web questionnaire [11], completed up to four times by over 200,000 participants, and from the first repeat of the entire baseline assessment in around 20,000 participants [12]. Over the coming months and years, further data will become available from: a range of biochemical assays and genome-wide genotyping of baseline samples from all participants; Web-based questionnaires to assess specific characteristics in more detail (e.g., cognitive function, occupational history); and, in subsets of 100,000 participants, collection of data from physical activity monitors and multi-modal imaging (Table 3).

**Table 3:** Current and planned future data available in the UK Biobank resource.

Data type	Number of participants	Details	Date of data acquisition	Date first available for research‡
**Baseline assessment**	Whole cohort	Questionnaire, physical measures, samples (see Table 2); haematological assays done on fresh blood samples	2006–2010	Q2 2012
**Repeat of baseline assessment**	20,000–25,000	As above every few years, to allow correction for regression dilution due to measurement error and within person fluctuations in exposure levels [12].	2013–	Q3 2013
**Biochemical assays (of baseline samples)**	Whole cohort	Biomarkers with known disease associations (e.g., lipids for vascular disease), diagnostic value (e.g., HbA_1c_ for diabetes), or ability to characterize phenotypes not otherwise well assessed (e.g., renal and liver function tests).	2014–2015	2015
**Genotyping (of baseline samples)**	Whole cohort	Dense genotyping chip with >800,000 markers including: approximately 250,000 SNPs in a whole-genome array; approximately 200,000 markers covering CNV, loss of function, insertions, deletions, and previously identified risk factor or disease associations; approximately 150,000 exome markers covering a high proportion of non-synonymous coding variants with allele frequency >0.02%.	2013–2015	2015
**Dietary Web questionnaire**	210,000	Automatically coded dietary recall questionnaire, providing estimates of nutrient intake. 80,000 respondents completed it ≥ three times.	2011–2012	Q2 2013
**Other Web questionnaires**	350,000 to be approached	Participants invited by email to provide additional information via Web questionnaires about exposures (e.g., occupation) and health outcomes (cognitive function, depression) that are not readily identified from health record linkages.	2014–	2015
**Accelerometry**	100,000	Wrist-worn tri-axial accelerometers record information on type, intensity, and duration of physical activity.	2013–2015	2015
**Multimodal imaging**	100,000	MRI brain, heart, and abdomen (for lipid distribution); ultrasound of carotid arteries; whole body DXA scan of bones and joints	Pilot phase: 2014–2015 Main phase: 2016–2019	2015
**Health record linkage**	Whole cohort			
Death registrations		ICD-coded cause specific mortality	2006–	Q2 2013
Cancer registrations		ICD-coded cancer diagnoses	1971–*	Q2 2013
Hospital inpatient episodes		ICD-coded diagnoses, OPCS-coded procedures	1997–*	Q4 2013
Hospital outpatient episodes		Limited ICD and OPCS coding	2003–*	2015
Primary care		Read-coded information including diagnoses, measurements, referrals, prescriptions	Variable	2015
Other		UK Biobank will obtain data from national mental health care, residential history, laboratory and disease audit datasets and is considering the value of further linkages (e.g., imaging, cancer screening, dental).	Variable	Not yet determined
**Adjudicated health outcomes**	Whole cohort	Expert-led confirmation and subclassification of outcomes in a range of disease areas, including cancer, diabetes, heart disease, stroke, mental health, musculoskeletal, respiratory, neurodegenerative, and ocular disorders.		2015

Hb: haemoglobin; SNPs: single nucleotide polymorphisms; CNV: copy number variations; MRI: magnetic resonance imaging; DXA: dual-energy X-ray absorptiometry; ICD: International Classification of Diseases; OPCS: Office of Population Censuses and Surveys Classification of Interventions and Procedures

^‡^ Future dates are estimated. Data available may be all or part of the relevant dataset.

* available from an earlier date from health record systems in Scotland

### Data from Longitudinal Follow-Up for Health-Related Outcomes

Follow-up is conducted chiefly through linkages to routinely available national datasets. Data are already available on over 8,500 deaths, over 75,000 prevalent and incident cancers, and over 600,000 hospital admissions, while linkages are planned to a range of other datasets, including primary care, cancer screening data, and disease-specific registers. In addition, to reduce misclassification and increase biological specificity of health outcomes, UK Biobank is developing methods for accurate identification and detailed phenotyping of outcomes in a range of disease areas. Initial ascertainment of outcomes with electronic and semi-automated sources will be supplemented by more intensive methods (e.g., retrieval of case records, imaging data, or banked tissue samples) for validation and subclassification (Table 3).

## Online Open Access to Researchers

Many cohort studies have mechanisms for sharing data with external researchers on a collaborative basis, but relatively few have arrangements for open access to the data without any need for collaboration, and even fewer have been established from the outset with the intention of making the entire resource available to the global research community. The development of open access arrangements for data from cohort studies is an important step in maximising their impact with respect to scientific publications, policy making, and understanding of health and disease. Examples of resources whose impact has been enhanced in this way include the UK 1958 birth cohort study [13] and the Australian 45 and Up cohort study [14].

UK Biobank aims to encourage and provide as wide access as possible to its data and samples for health-related research in the public interest by all bona fide researchers from the academic, charity, public, and commercial sectors, both in the UK and internationally, without preferential or exclusive access for any user. UK Biobank’s publicly available Data Showcase (http://www.ukbiobank.ac.uk/) presents the univariate distributions and methods used for collection of all the variables available for health-related research, enabling potential research users to explore what data are available and plan research applications.

An online access process, launched in April 2012, aims to be fair, transparent, and streamlined. Applications for data only are approved so long as the proposed research is in the public interest and the data required are, or will become, available. Applications involving the use of depletable samples or requiring participant re-contact are subject to a more rigorous process of scrutiny and scientific review. Following initial assessment by the executive team, all applications are assessed and either approved or rejected (with right of appeal) by an independent Access Subcommittee. Advice is sought on any applications raising potential ethical issues from both the University of Oxford’s Ethox Centre and the Ethics and Governance Council. Only de-identified data are provided to researchers, who must sign a material transfer agreement, undertaking not to attempt to identify any participant, to keep the data secure, and to use it only for the purposes of the approved research. Researchers must also undertake to publish their results and to return details of their methods, derived data, and/or sample assay results for incorporation into the UK Biobank dataset so that they can be made available to other approved researchers (see www.ukbiobank.ac.uk/scientists/ for details). UK Biobank encourages, but does not mandate, publication of results of research based on the resource in open access journals. Ensuring that the resource and its access arrangements are widely communicated is an important task, requiring a dedicated communications team to manage UK Biobank’s website, scientific meetings, and other methods for communication with the scientific community, including emails, newsletters, and other social media. In the first two years after the launch of open access to UK Biobank, over 1,000 researchers successfully registered, and over 200 applications were submitted (see www.ukbiobank.ac.uk/approved-research/ for a summary of research that is currently underway). Over 80% of registered researchers were from the UK and over 95% from academic rather than commercial institutions. Approximately 85% of applications were for data only, with few as yet requesting use of samples or participant re-contact. UK Biobank has now started to receive notifications of submitted abstracts and manuscripts based on the first few completed research projects. UK Biobank reviews its access procedures regularly, revising them in the light of experience and user feedback to make the process as streamlined as possible while remaining consistent with participant consent.

## Running UK Biobank

Success so far in developing and enhancing the resource has relied on public willingness to participate in prospective research studies; close engagement with funders, government health departments, and the UK National Health Service; extensive consultation with the public, scientists, and a wide range of regulatory, legal, and ethics bodies; and the development of cost-effective and efficient methodological approaches. The most significant challenges to be overcome are the implementation of scientifically rigorous processes on a very large scale, sustaining the funding required to ensure the benefits of the resource are fully realised, obtaining approvals from multiple regulatory bodies in a frequently changing political and healthcare environment, and ensuring as wide as possible communication of the non-preferential, open access nature of the resource.

### Interactions with Participants

Participant recruitment, retention, and engagement with enhancement projects has benefited from the willingness of very large numbers of British people to take part in observational research without the prospect of direct personal gain [15]. Participants spent an average of about two and a half hours at the recruitment visit. All gave broad consent to use of their anonymised data and samples for any health-related research, to be re-contacted for further substudies, and for UK Biobank to access their health-related records. Large subsets have subsequently completed Web-based questionnaires, agreed to wear a physical activity monitor, and repeated the entire baseline assessment. Of those who attended the first repeat assessment visit and provided feedback, 92% reported that they would be willing to travel for up to two hours for an imaging assessment visit lasting half a day. UK Biobank keeps its participants involved through providing progress updates via its website, with annual newsletters, and through its dedicated Participant Resource Centre (PRC), enabling them to continue to support the project and participate in research over the years ahead.

### Interactions with Funding Bodies

Having established UK Biobank as a charitable company over a decade ago, the UK Medical Research Council and Wellcome Trust have provided the vast majority of its funding so far. These major funders have had the long-term vision to continue to invest substantially in its ongoing development as a global research resource, coordinating both the scientific review of major proposals for developments to the resource and contributions from other funding bodies, including the Department of Health, Scottish and Welsh Governments, North West Development Agency, British Heart Foundation, and Diabetes UK. Long-term funding is not guaranteed, but depends on UK Biobank working in close partnership with its funders towards the common goal of facilitating high-quality, cost-effective research that will improve the public’s health. Crucial to this partnership is provision and joint discussion of regular updates on progress against challenging milestones, new strategic goals, scientific opportunities, financial plans, and use of the resource to generate new scientific knowledge.

### Interactions with the UK’s Publicly Funded National Health Service

Participant recruitment relied on invitations being mailed to 9 million people whose contact details were obtained from National Health Service (NHS) central registers. Large-scale epidemiological studies in the UK benefit from the fact that 98% of the population is registered with the NHS, which keeps detailed records on all of them from birth to death. Linkages to NHS datasets provide the principal means of follow-up for health-related outcomes.

### Industrial Scale, Centralised Processes

A key step in achieving the cost-effective recruitment, characterisation, and follow-up of 500,000 participants was the creation of an executive and advisory team with complementary scientific and management skills and a coordinating centre dedicated to the generation of a resource for the scientific community. This facilitated the development of a centralised infrastructure, bespoke information technology (IT) systems, and industrial approaches to collection and processing of data and samples. For example, inviting potential participants via individual general-practice groupings (an approach used by smaller UK population-based studies) would have been impractical for a study of UK Biobank’s scale, so appropriate approvals were obtained to allow direct mailing of invitations using contact details held centrally by the NHS. The recruitment process itself was coordinated centrally, with up to six assessment centres being active at any one time during the recruitment phase. Staffing and equipment needs were carefully configured to ensure the smooth flow of around 100 participants per day through each assessment centre for six days per week. Biological samples were also processed and handled centrally, requiring the development of bespoke laboratory information management and automated robotic systems to facilitate rapid, error-free sample storage in, and extraction from, the freezers (at rates of up to 1,500 samples per day) according to particular sample and participant characteristics [16]. Each step of the recruitment, assessment, and sample handling process was first piloted, modified as necessary and monitored centrally, using statistical methods to identify potential performance issues. Similar industrial-scale, centralised processes have been or are being developed for the repeat assessment and imaging visits.

### Governance Structure

UK Biobank’s Board of Directors has overall responsibility for its direction and management. An Executive Management Team, with epidemiology, clinical, management, laboratory, legal, and communications expertise, oversees the development and day-to-day management of the resource and is responsible for the staff working on the study, most of them based at its coordinating centre near Manchester, with others at the Universities of Oxford, Edinburgh, Cardiff, and London. The executive team receives guidance from a Steering Committee of leading UK scientists, supported by specialist working groups advising on baseline data collection, enhanced phenotyping, follow-up and outcomes adjudication, and an international perspective is provided by an International Scientific Advisory Board (see S1 Consent Form and www.ukbiobank.ac.uk/governance/). This governance structure has facilitated effective working between scientific and management disciplines, allowing UK Biobank to respond to advice from a wide network of scientists on the most scientifically valuable design and development of the resource, with project management and implementation being the responsibility of UK Biobank’s Executive Management Team and dedicated staff.

### Robust Ethics and Governance Framework

UK Biobank has consulted widely not only with the scientific community but also with the public, its participants, and other interested parties [17,18]. This has informed the development of its Ethics and Governance Framework, which lays out its principles and policies [19], as well as its access procedures [20]. UK Biobank’s research ethics committee and Human Tissue Authority research tissue bank approvals mean that researchers wishing to use the resource do not need separate ethics approval (unless re-contact with participants is required). An independent Ethics and Governance Council oversees adherence to the Ethics and Governance Framework and provides advice on the interests of research participants and the general public in relation to UK Biobank.

In keeping with the informed consent given by its participants, UK Biobank does not generally provide feedback to individual participants about information derived from analyses of data or samples made following their assessment visits. Participants receive limited individual feedback in two areas. First, they receive a summary of standard measures (e.g., blood pressure, body mass index) at the end of each assessment visit and are encouraged to seek medical advice for results outside the normal range. Second, potentially serious incidental findings (i.e., those likely to threaten life span or have a major impact on quality of life) observed by study staff during these assessments (e.g., possible melanoma on exposed areas of skin) are brought to the attention of participants with encouragement to contact a relevant health professional. Similar feedback is occurring in the imaging substudy, with participants and their general practitioners informed of potentially serious incidental findings noticed by radiographers and confirmed by formal radiologist review. In addition, the overall findings and implications of results that derive from research using the UK Biobank resource are made available to researchers, participants, and the wider community so that they can influence public health strategies.

### Interactions with Regulatory Bodies

The wide consultation, rigorous Ethics and Governance Framework, and Ethics and Governance Council oversight role have been essential in paving the way for UK Biobank to accomplish obtaining the multiple ethical and regulatory approvals required for participant recruitment, sample and data storage, linkages to routine health care data, enhancement studies, and the provision of access to data and samples for approved researchers. Substantial amounts of time, resources, patience, tenacity, and evidence of feasibility and/or acceptability from smaller scale pilot studies have also been required to provide regulatory bodies with the reassurance that they need of UK Biobank’s rigorous approach and commitment to protecting the interests of its participants within an acceptable legal and ethical framework.

## Conclusions

The key lessons learned from establishing UK Biobank are that such large-scale studies require not only a clear scientific focus but also streamlined governance; effective working between academic and management disciplines; centralised infrastructure with industrial approaches to collection and processing of data and samples; close partnership with major funders; a wide network of scientific advisors; high-quality, pragmatic legal and ethical advice; and widespread public support [21]. The resource is now facilitating research by scientists from around the world who wish to investigate how different diseases are caused by the combination of lifestyle, environment, and genes, leading to improvements in prevention, diagnosis, and treatment. Perhaps unsurprisingly, early use has been mainly, but not exclusively, by UK-based scientists. A major aim for the immediate future is to encourage applications from outside the UK. To facilitate this, UK Biobank is further developing its communications strategy to increase awareness of the resource and its access procedures worldwide.

## Supporting Information

S1 Consent Form (PDF)

S1 Text UK Biobank Committees and Working Groups. (PDF)
