# Supplementary material for: UK Biobank: An Open Access Resource for Identifying the Causes of a Wide Range of Complex Diseases of Middle and Old Age
Source: PLoS Med. 2015 Mar 31;12(3):e1001779. doi: 10.1371/journal.pmed.1001779 (PMC4380465; doi:10.1371/journal.pmed.1001779)
Supplement: S1 Consent Form — (PDF) [file pmed.1001779.s001.pdf]

Consent Form: UK Biobank

Assessment centre number: [INSERT CENTRE NUMBER]
Participant identifier: [INSERT PARTICIPANT IDENTIFIER]

The purpose of UK Biobank is to set up a resource that can support a diverse range of research intended to improve the prevention, diagnosis and treatment of illness, and the promotion of health throughout society. Thank you for reading the Information Leaflet, and asking any questions that you might have had. If you would like to participate, please respond to each of the following questions on the touch-screen and then sign the computer pad.

I have read and understand the Information Leaflet, and have had the opportunity to ask questions.	**I agree**
I understand that my participation is voluntary and that I am free to withdraw at any time without giving any reason.	**I agree**
I understand that I may be re-contacted by UK Biobank (e.g. to answer some more questions and/or attend another assessment visit), but this is optional.	**I agree**
I give permission for access to my medical and other health-related records, and for long-term storage and use of this and other information about me, for health-related research purposes (even after my incapacity or death).	**I agree**
I give permission for long-term storage and use of my blood and urine samples for health-related research purposes (even after my incapacity or death), and relinquish all rights to these samples which I am donating to UK Biobank.	**I agree**
I understand that none of my results will be given to me (except for some measurements during this visit) and that I will not benefit financially from taking part (e.g. if research leads to commercial development of a new treatment).	**I agree**
I agree to take part in UK Biobank.	**I agree**

[INSERT PARTICIPANT NAME]	[INSERT DATE]	[INSERT PARTICIPANT SIGNATURE]
**Volunteer name**	**Date**	**Signature**

[INSERT STAFF MEMBER NAME]	[INSERT DATE]
**Staff member name**	**Date**

For further information about UK Biobank, please call free of charge on 0800-0-276-276 or look at the project website at www.ukbiobank.ac.uk
