# Supplementary material for: UK Biobank: An Open Access Resource for Identifying the Causes of a Wide Range of Complex Diseases of Middle and Old Age
Source: PLoS Med. 2015 Mar 31;12(3):e1001779. doi: 10.1371/journal.pmed.1001779 (PMC4380465; doi:10.1371/journal.pmed.1001779)
Supplement: S1 Text — (PDF) [file pmed.1001779.s002.pdf]

UK Biobank Committees and Working Groups

UK Biobank Board

Chair: Prof Sir Mike Rawlins (University of Newcastle)

Ms Genny Kiff (Wellcome Trust)

Ms Simone Bayes (Department of Health)

Mr Jonathan Tross (independent member)

Ms Tara Camm (independent member)

Prof Sir Andy Haines (LSHTM)

Prof Andrew Hattersley (University of Exeter)

Prof Bill Ollier (University of Manchester)

Prof Patrick Vallance (GSK)

Mr Ian Viney (MRC)

Dr Jimmy Whitworth (Wellcome Trust)

Secretary: Mr Jonathan Sellors

Previous Chair: Sir Alan Langlands (Higher Education Funding Council, England)

Previous members: Prof Danny Altman (Wellcome Trust); Prof Sir John Bell (University of Oxford); Dr Peter Craig (Chief Scientist Office, Scotland); Dr Wendy Ewart (MRC); Prof Mike Pringle (University of Nottingham); Dr Pat Goodwin (Wellcome Trust); Ms Jane Lee (MRC); Dr Barbara Skene (Wellcome Trust); Dr Alison Spaull (Chief Scientist Office, Scotland); Mr Marc Taylor (Department of Health)

Previous Board Secretary Mr Andrew Moberly

Steering Committee

Principal Investigator/Chair: Prof Sir Rory Collins (University of Oxford/UK Biobank)

Prof John Danesh (University of Cambridge)

Prof Paul Elliott (Imperial College London)

Dr John Gallacher (University of Cardiff)

Prof Jane Green (University of Oxford)

Prof Paul Matthews (Hammersmith Hospital)

Prof Jill Pell (University of Glasgow)

Dr Tim Sprosen (University of Oxford)

Secretariat: Dr Naomi Allen (University of Oxford/UK Biobank); Prof Cathie Sudlow (University of Edinburgh/UK Biobank); Dr Tim Peakman (UK Biobank);

Previous members: Prof Dame Valerie Beral (University of Oxford); Prof Paul Burton (University of Leicester); Prof Alan Silman (University of Manchester)

Subgroups of Steering Committee

Longitudinal Follow-up

Chair: Prof Mike Pringle (Nottingham)

Deputy Chair: Dr Tim Sprosen (London/Oxford)

Prof Sir Rory Collins (Oxford)

Prof Frank Sullivan (Dundee)

Dr Mark McGilchrist (Dundee)

Prof Ronan Lyons (Swansea)

Prof Azeem Majeed (London)

Secretariat: Prof Cathie Sudlow (University of Edinburgh/UK Biobank

Previous secretariat: Dr Giok Ong (University of Oxford/UK Biobank)

Outcomes (speciality in italics)

Chair: Prof John Danesh (*cardiovascular disease*)

Prof Carol Brayne (*neurodegenerative disorders*)

Prof Nish Chaturvedi (*diabetes*)

Prof Sir Rory Collins (*epidemiology*)

Prof Ian Ford (*biostatistics*)

Dr John Gallacher (*mental health*)

Prof Harry Hemingway (*cardiovascular disease*)

Prof Alan Silman (*musculoskeletal disorders*)

Dr Terence O'Neill (*musculoskeletal disorders*)

Prof Cathie Sudlow (*stroke*)

Prof Simon Wessely (*psychiatric conditions*)

Prof Matthew Hotopf (*psychiatric conditions*)

Prof David Strachan *(respiratory conditions)*

Prof David Lomas *(respiratory conditions)*

Dr Michael Chapman *(cancer)*

Dr Henrik Moller *(cancer)*

Secretariat: Prof Cathie Sudlow (University of Edinburgh/UK Biobank); Dr Naomi Allen (University of Oxford; UK Biobank)

Previous secretariat: Dr Giok Ong (University of Oxford/UK Biobank); Dr Tim Sprosen (University of Oxford); Dr Emanuele Di Angelantonio (Cambridge)

Enhancements

Chair: Prof Paul Elliott (London)

Prof Mark Caulfield (London)

Prof Sir Rory Collins (Oxford)

Dr John Gallacher (Cardiff)

Prof Alan Silman (Manchester)

Prof Nick Wareham (Cambridge)

Secretariat: Dr Ioanna Tzoulaki (London); Dr Naomi Allen (University of Oxford/UK Biobank); Prof Cathie Sudlow (University of Edinburgh; UK Biobank)

Imaging

Chair: Prof Paul Matthews (London)

Prof Jimmy Bell (London)

Prof Andrew Blamire (Newcastle)

Prof Sir Rory Collins (Oxford/UK Biobank)

Dr Tony Goldstone (London)

Dr Nick Harvey (Southampton)

Prof Alan Jackson (Manchester)

Dr Paul Leeson (Oxford)

Dr Karla Miller (Oxford)

Prof Stefan Neubauer (Oxford)

Prof Steffen Petersen (London)

Prof Stephen Smith (Oxford)

Secretariat: Dr Tim Peakman (UK Biobank); Mr Steve Garratt (UK Biobank); Dr Naomi Allen (University of Oxford/UK Biobank); Prof Cathie Sudlow (University of Edinburgh/UK Biobank)

Previous secretariat: Mr Paul Downey (UK Biobank)

Genotyping

Chair: Prof Peter Donnelly (Oxford)

Dr Jeff Barrett (Sanger Institute)

Dr Jose Bras (UCL)

Dr Adam Butterworth (Cambridge)

Prof Richard Durbin (Sanger Institute)

Prof Paul Elliot (Imperial)

Prof Ian Hall (Nottingham)

Prof John Hardy (UCL)

Prof Mark McCarthy (Oxford)

Prof Gil McVean (Oxford)

Prof Nazneen Rahman (Institute of Cancer Research)

Prof Nilesh Samani (Leicester)

Prof Martin Tobin (Leicester),

Prof Hugh Watkins (Oxford)

Secretariat: Dr Tim Peakman (UK Biobank)

Coordinating Centre (University of Manchester)

Ms Nicola Doherty

Mr Steve Garratt

Ms Kirsty Lomas

Ms Pamela Moore

Mr Grant Nash

Dr Tim Peakman

Health informatics research (University of Oxford)

Dr Ligia Adamska

Ms Victoria Allan

Dr Naomi Allen

Mr Bob Goodenough

Dr Martin Landray

Dr Mike Lay

Ms Helen Todd

Dr Alan Young

Data linkage and outcomes adjudication coordination (University of Edinburgh)

Ms Robin Flaig

Prof Cathie Sudlow

Dr Qiuli Zhang

Head of communications (University of Oxford)

Mr Andrew Trehearne

Participant Resource Centre (University of Cardiff)

Dr John Gallacher

Mrs Truda Bell

Ms Ana Koleva-Thompson

Mrs Maggie Gregory

Training and Monitoring Team (University of Glasgow)

Prof Jill Pell

Ms Barbara de Beul

Ms Jacquie Howe

Ms Margaret Webb

Ms Karen Johnson

Dr Giok Ong (University of Oxford & UK Biobank)

Ms Caron Paterson

International Scientific Advisory Board

Chair: Prof Bernard Keavney (Manchester, UK)

Prof Dame Valerie Beral (Oxford, UK)

Prof Peter Donnelly (Oxford, UK)

Prof Terry Dwyer (Melbourne, Australia)

Dr Mike Gaziano (Boston, USA)

Dr Prabhat Jha (Toronto, Canada)

Prof Hans-Ulrich Kauczor (Heidelberg, Germany)

Prof Michael Kidd (Adelaide, Australia)

Prof Mark Lathrop (Paris, France)

Dr Teri Manolio (Washington DC, USA)

Dr Giles Maskell (London, UK)

Prof Mads Melbye (Copenhagen, Denmark)

Prof Neil Risch (Palo Alto, USA)

Prof Dan Roden (Nashville, USA)

Prof Naveed Sattar (Glasgow, UK)

Dr Chris Wild (Lyon, France)

Previous members: Prof Paul Burton (Leicester, UK); Prof John Danesh (Cambridge, UK); Dr Silvia Franceschi (Lyon, France); Prof Susan Hankinson (Boston, USA); Dr Tom Hudson (Montreal, Canada); Prof Stephen MacMahon (Sydney, Australia); Prof Sir Richard Peto (Oxford, UK); Dr Michael Thun (Atlanta, USA)

Ethics & Governance Council (speciality and institution in parenthesis)

Chair: Prof Roger Brownsword (Medical jurisprudence; retired)

Dr Jonathan Hewitt (Gerontology; Portsmouth NHS Trust)

Prof Søren Holm (Bioethics; Manchester University)

Prof Nils Hoppe (Life Sciences Regulation; University of Hannover)

Prof Kate Hunt (Social policy; Glasgow)

Dr Sheelagh McGuinness (Ethics and Law; Keele University)

Ms Tracey Phillips (Public policy: Management consultant)

Mr Andrew Russell (retired)

Mrs Margaret Shotter (Research ethics)

Mr David Walker (Public policy)

Dr Susan E Wallace (Population and Public Health Sciences; University of Leicester)

Secretariat: Ms Adrienne Hunt (Wellcome Trust)

Previous Chairs: Prof Alastair Campbell (Ethics; University of Bristol); Prof Graeme Laurie (Medical jurisprudence, University of Edinburgh)

Previous members: Ms Andrea Cook; Ms Jayam Dalal; Baroness llora Finlay; Prof Erica Haimes; Prof Roger Higgs; Prof Ian Hughes; Dr Anneke Lucassen; Ms Clara MacKay; Prof Sheila McLean; Dr Roger Moore; Ms Hilary Newiss; Prof Martin Richards; Ms Sally Smith; Prof Sandy Thomas; Prof Paolo Vineis; Prof Heather Widdows; Prof Chris Wild

Access Subcommittee

Chair: Ms Tara Camm

Prof Sir Andrew Haines (London School of Hygiene and Tropical Medicine)

Prof Andrew Hattersley (University of Exeter)

Prof Sir Michael Rawlins (University of Newcastle)

Previous Science Committee (2003-5)

Chair: Prof Sir John Bell (Oxford)

Prof Dame Valerie Beral (Oxford)

Prof Paul Burton (Leicester)

Prof John Danesh (Cambridge)

Dr Richard Durbin (Cambridge)

Prof Paul Elliott (London)

Dr John Gallacher (Cardiff)

Prof Hilary Graham (Lancaster)

Prof Bernard Keavney (Newcastle)

Prof Stephen Palmer (Cardiff)

Prof Catherine Peckham (London)

Prof Jill Pell (Glasgow)

Prof Mike Pringle (Nottingham)

Prof John Todd (Cambridge)

Prof Alan Silman (Manchester)

Ms Madeleine Wang (Lay member)

Observers: Dr Alan Doyle (Wellcome Trust)*;* Prof Bill Ollier (University of Manchester); Ms Caroline Stone (MRC)

Secretariat: Dr John Newton (previous UK Biobank Chief Executive); Dr Tim Sprosen (previous UK Biobank Chief Scientist)

Previous working groups of Science Committee

Recruitment implementation

Chair: Prof Alan Silman (Manchester)

Prof Jane Armitage (Oxford)

Prof John Danesh (Cambridge)

Prof Richard Hobbs (Birmingham)

Dr Ron Hsu (Leicester)

Prof Gary MacFarlane (Manchester)

Dr Blair Smith (Aberdeen)

Paul Wallace (London)

Secretariat: Dr Tim Sprosen; Dr Tim Peakman and Mr Steve Walker (UK Biobank)

Recruitment principles

Chair: Stephen Palmer (Cardiff)

Prof Sube Banerjee (London)

Prof Raj Bhopal (Edinburgh)

Prof Sir Rory Collins (Oxford)

Dr John Gallacher (Cardiff)

Prof Richard Hobbs (Birmingham)

Prof Gary MacFarlane (Manchester)

Prof Frank Sullivan (Dundee)

Secretariat: Dr Tim Sprosen

Ethnic minorities

Chair: Prof Mark Caulfield (London)

Dr Raghib Ali (Oxford)

Dr Sube Banerjee (London)

Dr Gene Feder (London)

Dr Parmajit Gill (Birmingham)

Dr Ron Hsu (Leicester)

Dr Kamlesh Khunti (Leicester)

Dr Jaspal Kooner (London)

Dr Brenda Leese (Leeds)

Dr John MacLeod (Birmingham)

Secretariat: Dr Tim Sprosen (UK Biobank)

Questionnaire

Chair: Prof Dame Valerie Beral (Oxford)

Prof Cyrus Cooper (Southampton)

Dr John Gallacher (Cardiff)

Prof Hilary Graham (York)

Dr Bette Liu (Oxford)

Prof Catherine Peckham (London)

Prof Jill Pell (Glasgow)

Secretariat: Dr Tim Sprosen (UK Biobank)

Environment

Chair: Prof David Coggan (Southampton)

Dr Raymond Agius (Manchester)

Dr John Ayres (Aberdeen)

Dr Peter Blain (Newcastle)

Dr Robert Clarke (Oxford)

Dr Robin Fielder (Health Protection Agency)

Dr John Gallacher (Cardiff)

Dr Mark Nieuwenhuijsen (London)

Dr Marti Van-Tongeren (Manchester)

Secretariat: Dr Tim Sprosen (UK Biobank)

Diet

Chair: Prof Stephen Palmer (Cardiff)

Prof Sheila Bingham (Cambridge)

Prof Tim Key (Oxford)

Prof Ken Muir (Nottingham)

Secretariat: Dr Tim Sprosen (UK Biobank)

Cognition/psychological

Chair: Dr John Gallacher (Cardiff)

Prof Carol Brayne (Cambridge)

Dr Tony Kendrick (Southampton)

Dr Glyn Lewis (Bristol)

Dr John McBeth (Manchester)

Prof Peter McGuffin (London)

Prof Martin Prince (London)

Secretariat: Dr Tim Sprosen (UK Biobank)

Measurements

Chair: Prof Paul Elliott (London)

Prof Paul Burton (Leicester)

Prof Mark Caulfield (London)

Dr Robert Clarke (Oxford)

Dr Anna Hansell (London)

Prof Gary MacFarlane (Manchester)

Secretariat: Dr Tim Sprosen (UK Biobank)

Sample handling

Chair: Prof Paul Elliott (London)

Prof Mark Caulfield (London)

Dr Nick Davies (IBM Consulting)

Prof Anna Dominiczak (Glasgow)

Prof Mark McCarthy (Oxford)

Prof Bill Ollier (Manchester)

Prof Nilesh Samani (Leicester)

Dr Julian Sampson (Cardiff)

Secretariat: Dr Tim Peakman (UK Biobank)

Data Management

Chair: Prof Richard Durbin (Cambridge)

Dr Keith Cole (Manchester)

Prof Ronan Lyons (Swansea)

Prof Andrew Morris (Dundee)

Dr John Newton (UK Biobank)

Prof Jill Pell (Glasgow)

Prof Mike Pringle (Nottingham)

Dr Rhian Hughes (Keele)

Prof Alan Silman (Manchester)

Mr Neil Walker (Cambridge)

Draft Protocol Development Committee

Chair: Prof Tom Meade (LSHTM)

Prof Paul Burton (Leicester)

Dr Lon Cardon (Oxford)

Prof Nick Day (Cambridge)

Prof Anna Dominiczak (Glasgow)

Mr Mark Duman (Lay member)

Dr Rhydian Hapgood (Sheffield)

Prof Mark McCarthy (London)

Prof David Porteous (Edinburgh)

Prof Martin Prince (London)

Prof Ian Purves (Newcastle)

Dr Anne Richardson (Lay member)

Prof Alan Silman (Manchester)

Prof David Strachan (London)

Dr Ron Zimmern (Cambridge)

Protocol Writer: Dr Emily Banks

Secretariat/Funder Representatives: Dr Alan Doyle (Wellcome Trust); Mr Stephane Goldstein (MRC); Dr Peter Greenaway (Department of Health); Dr Frances Rawle (MRC); Ms Liz Shaw (Wellcome Trust)
